# 500 Meters Is a Result of 6-Minute Walk Test Which Differentiates Patients with High and Low Risk of Postoperative Complications after Lobectomy—A Validation Study

**DOI:** 10.3390/jcm10081686

**Published:** 2021-04-14

**Authors:** Tomasz Marjanski, Damian Wnuk, Robert Dziedzic, Marcin Ostrowski, Wioletta Sawicka, Witold Rzyman

**Affiliations:** 1 Thoracic Surgery Department, Medical University of Gdansk, Sklodowskiej-Curie 3A, 80-211 Gdansk, Poland; robert.dziedzic@gumed.edu.pl (R.D.); marcin.ostrowski@gumed.edu.pl (M.O.); witold.rzyman@gumed.edu.pl (W.R.); 2 Department of Physical Therapy, Medical University of Gdansk, Sklodowskiej-Curie 3A, 80-211 Gdansk, Poland; damian.wnuk@gumed.edu.pl; 3 Department of Anaesthesiology and Intensive Care, Medical University of Gdansk, Sklodowskiej-Curie 3A, 80-211 Gdansk, Poland; wioletta.sawicka@gumed.edu.pl

**Keywords:** lung cancer, lobectomy, complications, 6-min walking test

## Abstract

The 6-min walk test (6MWT) is a simple method of identifying patients with a high risk of postoperative complications. In this study, we internally validated the previously obtained threshold value of 500 m in the 6MWT as differentiating populations with a high and a low risk of postoperative complications after a lobectomy. Between November 2011 and November 2016, 624 patients who underwent a lobectomy and performed the 6MWT preoperatively entered this study. We compared the complication rates of two groups of patients—those who walked more than and those who walked less than 500 m. The patients who did not reach the distance of 500 m in the 6MWT were older (70 vs. 63 years *p* < 0.001), had worse pulmonary function tests (FEV1% 84 vs. 88 *p* = 0.041) and had a higher Charlson Comorbidity Index (*p* < 0.001). The patients who had a worse result in the 6MWT had a higher complication rate (52% vs. 42% *p* = 0.019; OR: 1.501 95% CI: 1.066–2.114) and a longer median postoperative hospital stay (7 vs. 6 days *p* = 0.010). In a multivariate analysis, the result of the 6MWT and pack-years proved to independently influence the risk of postoperative complications. This internal validation study confirms that 500 m is a result of the 6MWT which differentiates patients with a higher risk of postoperative complications and a prolonged hospital stay after a lobectomy.

## 1. Introduction

Surgical treatment of patients with lung cancer is, in most cases, associated with an inevitable deterioration of their general well-being. This fact requires clinicians to reliably obtain tools which will assess the perioperative risk. An appropriately presented perioperative risk enables sharing the decision about the treatment with patients and their families and offering them treatment tailored to their expectations.

Despite the commonly accepted guidelines on physiological qualification for lung cancer surgery [1,2], there is an ongoing discussion on their further optimization. These guidelines recommend assessment of the preoperative and calculation of predicted postoperative values of forced expiratory volume in the first second (FEV1) and diffusing capacity of the lungs for carbon monoxide (DLCO). If increased risk is detected on the basis of pulmonary function tests (PFT), then the patients should undergo the shuttle walk test, the stair climbing test or the cardiopulmonary exercise test (CPET). Low-tech tests are used in further assessment of middle- and low-risk patients [2]. The stair climbing test [3] and the shuttle walk test [4] have well-documented thresholds, enabling them to be incorporated into the guidelines. However, in the literature, there are conflicting (or not overlapping) thresholds of the stair climbing test [5], shuttle walk test [6], DLCO and FEV1 [7] or CPET [8], which raises the question about their exact clinical meaning.

Previously published cut-off values of the 6-min walk distance (6MWD) tended to allow appropriate stratification of the risk of postoperative complications. Patients who covered a distance shorter than 500 m had a significantly higher risk of postoperative complications (60.6% vs. 36.9%) and cardiopulmonary complications alone (43.9% vs. 24.5%), which resulted in a longer postoperative stay (7 vs. 6 days). Despite the clear clinical interpretation of the test, it was characterized by a relatively limited sensitivity (31.0%) and specificity (81.9%). This led us to perform internal validation of previously reported findings. The aim of the present study was to validate the result of 500 m in the 6-min walk test (6MWT) as an indicator of patients with an increased risk of postoperative complications after a lobectomy performed due to lung cancer. The study was performed in reproduced conditions of the same study center [9].

## 2. Material and Methods

This is an internal validation study verifying the findings of an initial report [9]. The protocols concerning the physiological qualification, surgical approach, anesthesia, perioperative management, recording of perioperative complications [9], description of 6MWT conditions [10] and 6MWD reference value calculation formula [11] were described before and did not change throughout the original study and this validation study.

**Figure 1:**
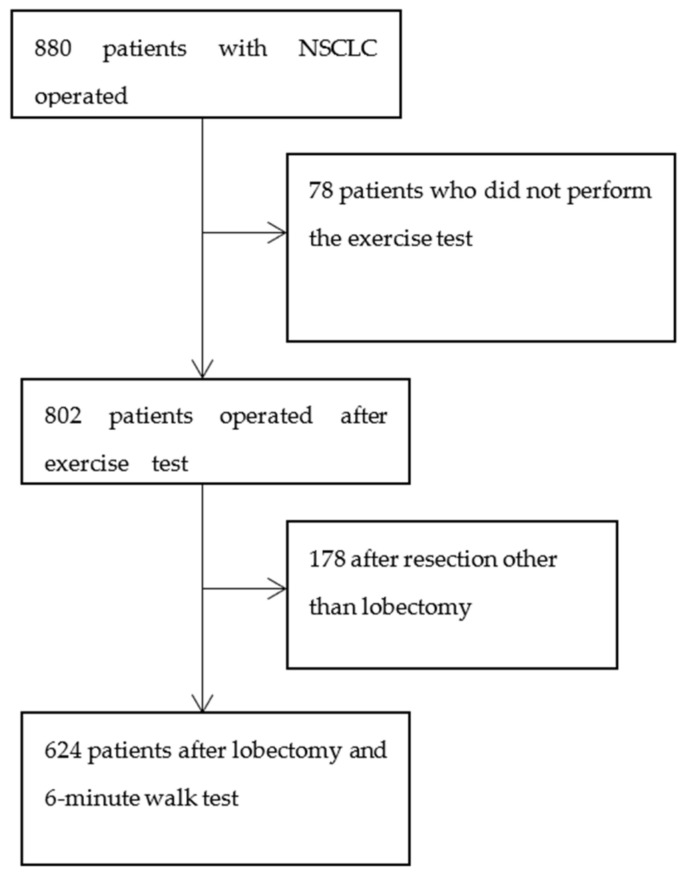
Study flow chart.

Between 1 November 2011 and 30 November 2016, 880 patients were operated on due to non-small cell lung cancer (NSCLC) in the Thoracic Surgery Department of the Medical University of Gdansk, Poland. We excluded 178 patients who had resections other than a lobectomy and 78 patients who did not perform the 6MWT due to medical conditions. A group of 624 patients who underwent a 6MWT on the day before the operation entered this study. The study flow chart is presented in Figure 1. This study was a retrospective analysis of the prospectively gathered data. The data were obtained from the Krajowa Baza Raka Pluca (National Registry of Lung Cancer)—a national, obligatory registry of all curative resections performed in patients with lung cancer in Poland. The registry data were matched with the supplemental data about the 6MWT.

The previously calculated cut-off values differentiated populations with a high and a low risk of postoperative complications after a lobectomy. We validated these cut-off values using the maximum area under the curve (AUC) of receiver operating curves (ROCs) to reassess the sensitivity and specificity of previously identified relations. The groups of patients (divided on the basis of the obtained 6MWD and percent of predicted value of 6-min walk distance (%6MWD)) were compared concerning clinical data, postoperative complications rate and length of postoperative stay. Unpaired data, characterized by a normal distribution, were compared with an unpaired t-test. In the case of a non-normal distribution, a Mann–Whitney U test was applied for comparing two unmatched samples. The χ^2^ test was used for categorical variables. The accepted level of statistical significance was *p* < 0.05. Odds ratios (ORs) were calculated with a 95% confidence interval (CI). Methods of logistic regression were utilized in multivariate analysis.

The study was approved by the institutional review board (NKBBN/88/2016).

## 3. Results

The group of 78 patients who did not perform the 6-min walk test and did not enter the analysis is characterized in Table 1.

The characteristics of the patients with the division based on the cut-off values are presented in Table 2.

The patients with a worse 6MWD and %6MWD were older and had a more significant smoking history, worse spirometry results and more concomitant diseases. Complications were more common in the group of patients with a 6MWD shorter than 500 m [52.4% vs. 42.3 OR 1.501 (95% CI 1.066–2.114)]. If the groups were divided by %6MWD, those who covered less than 100% had a higher rate of cardiopulmonary complications [41.9% vs. 34% OR 1.432 (95% CI 1.009–2.031)] and higher 30-day mortality (OR 5.793 (95% CI 1.114–30.128) *p* = 0.031]. The rates of other complications did not differ between the study groups. The length of the hospital stay was different in both study groups and is presented in Table 3.

In the study, we analyzed the occurrence of general complications depending on the result of the 6MWT and %6MWT. The value of 523 m was characterized by the highest area under the curve (AUC 0.579; 95% CI: 0.534–0.623; sensitivity 64.7%; specificity 48.8%; *p* < 0.001). The value of 109.5% for 6MWT% had the highest AUC of 0.549; 95% CI: 0.504–0.594; sensitivity = 49.4%; sensitivity 62.5%; *p* = 0.034.

In a univariate analysis of factors influencing the risk of general complications rate, we included FEV1 %, FEV1 dm3, FVC %, FVC dm3, pack-years, age, operative access, smoking, 6MWD (reference value (ref) 500 m), 6MWD (ref 100%) and CCI (ref 0–3). In the univariate analysis, FEV1%, operative access, age, Charlson Comorbidity Index (CCI), pack-years, FVC% and 6MWD were proved to influence the risk of complications (*p* < 0.05). After including them in stepwise logistic regression analysis, operative access (*p* = 0.018), age (ref 63) (*p* = 0.002) and FEV1 % (*p* = 0.001) proved to have an independent influence on the general complications rate. In an interactive model builder, 6MWD (ref 500 m) (*p* = 0.008) and pack-years (*p* < 0.001) were proved to independently influence the general complications rate.

## 4. Discussion

The main finding of our study is that the result of 500 m in the 6MWT sufficiently differentiates patients with different risk of complication rates after a lobectomy both in the validation study and original study (validation study: OR 1.501 95% CI 1.066–2.114 *p* = 0.019; original study OR 2.631 95% CI 1.423–4.880 *p* = 0.001) [9]. In addition, we found that a result below 100% in the 6MWT allowed for the identification of patients with a higher risk of cardiopulmonary complications (OR 1.432 95% CI 1.009–2.031 *p* = 0.045) and 30-day mortality (OR 5.793 95% CI 1.114–30.128 *p* = 0.031). These findings were not revealed in the original study. The low 30-day mortality (0.4%) in the original group disabled a comparison of this important factor.

Additionally, in the validation study, we did not confirm some previously noted correlations. In the previous study, the distance of 500 m allowed the identification of patients with a higher risk of cardiopulmonary complications, requirement for transfusion >2 units of blood and a longer hospital time. These were not confirmed in the validation study. The result of the 6MWT did not correspond with the length of the hospital stay.

The second most important finding is that, despite some discrepancies between the two studies, there is an observable, undisturbed trend toward a higher risk of all complications and mortality in groups of patients with worse results in the 6MWT.

The third significant finding is that ROC analysis provided similar AUCs in both studies with increased specificity and lowered sensitivity in the validation trial. Most importantly, we identified similar threshold values in the validation study (109.5% and 523 m) and in the original trial (100% and 500 m). We are aware that AUCs < 0.6 are not related to the best prediction models. However, in the original study, we identified a similar AUC. As a result of the non-optimal AUC, we previously postulated further studies on the topic. Both the original and validation studies supply convergent data from a large group of 877 patients.

Even good surgical candidates after the operation suffer from decreased exercise capacity [12], decreased quality of life [13] and often significant pain [14]. Furthermore, moderate- and high-risk candidates are threatened by a significant number of postoperative complications and mortality. Early identification of high-risk patients may result in the implementation of protocols lowering the number of postoperative complications and allowing the earliest treatment of life-threatening complications. Previous findings establish the role of high- [15] and low-tech tests [9,16,17,18] in the preoperative identification of patients with a high risk of perioperative complications. The 6-min walk test (6MWT) tends to be a good predictor of perioperative complications and early mortality after surgical treatment of patients with lung cancer [9,16,17,18,19,20]. In the majority of studies, the threshold values are between 300 and 500 m. Our studies consequently point out threshold values close to 500 m and 100%. Due to the mathematical (not arbitrary) method of establishing the threshold value and big study groups, we have accumulated evidence for the high clinical utility of this method. Moreover, this test tends to have the potential to identify patients with deteriorated long-term survival [21].

Traditionally used mathematical calculations of predicted postoperative (ppo) FEV1, DLCO and peak O_2_ consumption [1,2] are reliable tools to stratify the perioperative risk in candidates for lung cancer surgery. These pulmonary and cardiopulmonary tests are successfully used in risk stratification also in other surgical procedures: cardiac [22], cardiovascular [23] or abdominal [24]. The 6MWT is also a valuable method of preoperative risk assessment in oncological surgery [25], esophagectomy [26] or thymectomy [27]. The thresholds mentioned were similar (390–498 m) to those identified in patients before pulmonary resection. On the other hand, there are papers reporting that the 6MWT lacks predictive value in identifying the risk of pulmonary complications after abdominal surgery [28]. We believe that on the basis of this report and previous reports, we may now consider including the 6MWT into the algorithms of qualification for radical treatment of lung cancer patients.

Still, PFTs may pose a difficult task to perform for a certain group of patients. Some of the patients who are senile [29], tracheostomy patients or patients with hemoptysis, significant bronchial secretions, oral lesions or bleeding [30] cannot be appropriately evaluated with this diagnostic tool. Every day, thoracic oncologists are faced with patients with objectively poor PFTs and a conflictingly good exercise capacity and quality of life. These high-risk patients who are seeking treatment deserve a precise assessment of their perioperative risk, especially in the era of forthcoming promising treatment strategies such as sublobar resections, stereotactic body radiotherapy or radiofrequency ablation. We believe that such submaximal tests as the 6MWT may be easy and well tolerated, still providing a significant message.

Patients who are unable to complete or even start the 6MWT are a potentially vulnerable group. In the original study, there were 20.4% of those patients with a 49% complication rate and 7.6% 90-day mortality. Increased mortality (15.6%) was also observed in other studies concentrating on patients who did not manage to complete the stair climbing test [31]. In the validation study, there were only 8.8% of patients who did not have the test performed, which is a result of the optimization of qualification for surgery. In our study, the mortality and morbidity remained low also in patients who did not undergo the test.

The COVID-19 pandemic has led to reduced access to highly specialized diagnostic and therapeutic procedures. A survey conducted by ESTS showed that during periods of high SARS-CoV-2 transmission, 1/3 of patients do not have access to all the necessary preoperative assessment tools [32]. Due to the unpredictable limitations in availability, clinicians may need to select patients for treatment based on incomplete data. Current guidelines do not include low-tech tests (e.g., 6MWT, shuttle walk test or stair climbing test) as individual tools for assessing the risk of resection. They cannot be used for an unambiguous and independent risk assessment by qualification for treatment. However, these tests may be performed without any additional resources and involve only one member of medical staff. These tests may be performed in a medical mask if there is such a necessity. Under extreme circumstances, spirometry and CPET may be unavailable. However, we cannot recommend exclusive use of the 6MWT instead of routine methods in the COVID-19 pandemic.

There is area for future studies. Perhaps including other factors such as nutrition may increase the clinical value of the implemented low-tech tests. We postulate that easier tests and uncomplicated algorithms are more commonly reproduced in daily practice.

There are limitations which diminish the scientific value of this study. First of all, this study has a partly retrospective character. The effect of the limited power of retrospective analysis is somehow compensated by the repeated consecutive character of validation analysis. We did not incorporate the DLCO, CPET or specific cardiologic data due to a lack of these values in the national registry. These data are required components of the evaluation of fitness before lung cancer surgery. Incorporation of them into our analysis would improve it. We also did not correlate the result of the 6MWT with DLCO or CPET. Interpretation of the results of the study must include seemingly conflicting findings. Patients who have poorer results in the 6MWT in meters and in the percent of the reference value have constant trends toward higher mortality and higher complication rates. However, some values (general complication rate and 30-day mortality) may be confirmed by either result in meters or in percent. We believe that in general, the worse the result, the higher the risk of the operation. We decided to present conclusions on the basis of results in meters only.

## 5. Conclusions

This internal validation study confirms that 500 m is a result of the 6-min walk test which differentiates patients with a higher risk of postoperative complications and a prolonged hospital stay after a lobectomy.

## Figures and Tables

**Table 1:** Characteristics of patients who did not perform the 6-min walk test.

Smoking	92.0%
Age	64 (±6)
Pack-years	40 (±17.35)
FEV1 [dm^3^]	2.28 (±0.79)
FEV1%	88 (±20.13)
FVC [dm^3^]	3.09 (±0.91)
FVC%	96.5% (±21.16)
Complications rate	17.9%
Hospital mortality	1 (1.12%)
30-day mortality	0 (0.0%)
90-day mortality	3 (3.4%)
Median postoperative stay [days]	7 (±6.16)

FEV1—forced expiratory volume, FEV1%—percent of predicted value of forced expiratory volume, FVC—forced vital capacity, FVC%—percent of predicted value of forced vital capacity.

**Table 2:** Patient characteristics. Patients are divided into two groups depending on the 6-min walk distance (6MWD) and percent of predicted value of 6-min walk distance (%6MWD). The cut-off values are 500 m and 100%, respectively.

Clinical Feature	<500 m *n* = 191	≥500 m *n* = 433	*p*	<100% *n* = 191	≥100% *n* = 433	*p*
Smoking	172 (90.1%)	405 (93.5%)	0.129	180 (94.2%)	397 (91.7%)	0.265
Pack-years	40 ± 16.6	35 ± 19.2	0.713	40 ± 18.4	35 ± 18.3	0.002
Age	70 ± 6.8	63 ± 8.5	<0.001	64 ± 9.9	65 ± 7.8	0.151
FEV1	1.97 ± 0.6	2.37 ± 2.39	<0.001	2.34 ± 0.8	2.21 ± 2.4	0.379
FEV1%	84 ± 20.3	88 ± 20.5	0.041	81 ± 19.8	90 ± 20.3	<0.001
FVC	2.79 ± 5.2	3.39 ± 0.9	<0.001	3.37 ± 5.1	3.16 ± 0.9	0.096
FVC%	94 ± 19.7	100 ± 19.2	0.003	93 ± 18.7	101 ± 19.5	0.002
6MWD	445 ± 65.4	567 ± 52.8	<0.001	469.5 ± 101.1	558 ± 65.4	<0.001
6MWD%	95.47 ± 18.8	110.49 ± 12.1	<0.001	92.8 ± 13.5	112.66 ± 10.0	<0.001
VATS	51 (26.7%)	150 (34.7%)	0.05	53 (27.7%)	148 (34.2%)	0.113
Tis	3 (1.6%)	8 (1.9%)	0.793	2 (1.0%)	9 (2.1%)	0.357
pIA	64 (33.5%)	143 (33.0%)	0.996	65 (34.0%)	142 (32.8%)	0.850
pIB	52 (27.2%)	116 (26.8%)	0.988	46 (24.1%)	122 (28.2%)	0.247
pIIA	24 (12.6%)	67 (15.5%)	0.311	20 (10.5%)	71 (16.4%)	0.046
pIIB	23 (12.0%)	42 (9.7%)	0.409	29 (15.2%)	36 (8.3%)	0.012
pIIIA	23 (12.0%)	49 (11.3%)	0.839	26 (13.6%)	46 (10.6%)	0.309
pIIIB	0 (0%)	2 (0.5%)	0.344	1 (0.5%)	1 (0.2%)	0.558
pIV	2 (1.0%)	0 (0%)	0.034	2 (1.0%)	0 (0%)	0.034
CCI 0–3	61 (38.1%)	249 (66.6%)	<0.001	81 (51.9%)	229 (60.6%)	0.066
CCI 4+	99 (61.9%)	125 (33.4%)	<0.001	75 (48.1%)	149 (39.4%)	0.066

6MWD—6-min walk distance, 6MWD%—percent of predicted value of 6-min walk distance, CCI—Charlson Comorbidity Index, FEV1—forced expiratory volume, FEV1%—percent of predicted value of forced expiratory volume, FVC—forced vital capacity, FVC%—percent of predicted value of forced vital capacity, VATS—video-assisted thoracic surgery.

**Table 3:** Postoperative complications. Patients are divided into two groups depending on the 6MWD and %6MWD. The cut-off values are 500 m and 100%, respectively.

Clinical Feature	<500 m *n* = 191	≥500 m *n* = 433	*p*	OR (95% CI)
Complication rate	100 (52.4%)	183 (42.3%)	0.019	1.501 (1.066–2.114)
Cardiopulmonary complications rate	76 (39.8%)	149 (34.4%)	0.198	1.260 (0.887–1.790)
Atrial arrythmia	33 (17.3%)	63 (14.6%)	0.385	1.227 (0.774–1.944)
Persistent air leak	16 (8.4%)	43 (9.9%)	0.541	0.829 (0.455–1.513)
Atelectasis requiring aspiration	24 (12.6%)	45 (10.4%)	0.426	1.239 (0.731–2.100)
Transfusion of >2 units of blood	7 (3.7%)	7 (1.6%)	0.121	2.315 (0.801–6.695)
Drainage time	4 (0–25)	4 (1–25)	0.506	-
Hospital stay	7 (0–102)	6 (0–83)	0.010	-
30-day mortality	4 (2.1%)	3 (0.7%)	0.209	3.066 (0.679–13.834)
90-day mortality	6 (3.1%)	10 (2.3%)	0.586	1.372 (0.491–3.830)
**Clinical feature**	**<100%** ***n* = 191**	**≥100%** ***n* = 433**	***p***	**OR (95% CI)**
Complication rate	96 (50.3%)	187 (43.2%)	0.102	1.329 (0.945–1.871)
Cardiopulmonary complications rate	80 (41.9%)	145 (33.5%)	0.045	1.432 (1.009–2.031)
Atrial arrythmia	26 (13.6%)	70 (16.2%)	0.416	0.817 (0.503–1.329)
Persistent air leak	22 (11.5%)	37 (8.6%)	0.244	1.393 (0.798–2.433)
Atelectasis requiring aspiration	27 (14.1%)	42 (9.7%)	0.106	1.533 (0.914–2.570)
Transfusion of >2 units of blood	7 (3.7%)	7 (1.6%)	0.121	2.315 (0.801–6.695)
Drainage time	5 (0–25)	7 (0–102)	0.005	-
Hospital stay	4 (1–25)	6 (0–83)	<0.001	-
30-day mortality	5 (2.6%)	2 (0.5%)	0.031	5.793 (1.114–30.128)
90-day mortality	8 (4.2%)	8 (1.8%)	0.102	2.322 (0.858–6.283)

6MWD—6-min walk distance, 6MWD%—percent of predicted value of 6-min walk distance, CCI—Charlson Comorbidity Index, FEV1—forced expiratory volume, FEV1%—percent of predicted value of forced expiratory volume, FVC—forced vital capacity, FVC%—percent of predicted value of forced vital capacity, VATS—video-assisted thoracic surgery.

## Data Availability

Data are available on request due to restrictions, e.g., privacy or ethical.
